# Covariance among Multiple Health Risk Behaviors in Adolescents

**DOI:** 10.1371/journal.pone.0098141

**Published:** 2014-05-23

**Authors:** Kayla de la Haye, Elizabeth J. D'Amico, Jeremy N. V. Miles, Brett Ewing, Joan S. Tucker

**Affiliations:** RAND Corporation, Santa Monica, California, United States of America; Oregon Health & Science University, United States of America

## Abstract

**Purpose:**

In a diverse group of early adolescents, this study explores the co-occurrence of a broad range of health risk behaviors: alcohol, cigarette, and marijuana use; physical inactivity; sedentary computing/gaming; and the consumption of low-nutrient energy-dense food. We tested differences in the associations of unhealthy behaviors over time, and by gender, race/ethnicity, and socioeconomic status.

**Methods:**

Participants were 8360 students from 16 middle schools in California (50% female; 52% Hispanic, 17% Asian, 16% White, and 15% Black/multiethnic/other). Behaviors were measured with surveys in Spring 2010 and Spring 2011. Confirmatory factor analysis was used to assess if an underlying factor accounted for the covariance of multiple behaviors, and composite reliability methods were used to determine the degree to which behaviors were related.

**Results:**

The measured behaviors were explained by two moderately correlated factors: a ‘substance use risk factor’ and an ‘unhealthy eating and sedentary factor’. Physical inactivity did not reflect the latent factors as expected. There were few differences in the associations among these behaviors over time or by demographic characteristics.

**Conclusions:**

Two distinct, yet related groups of health compromising behaviors were identified that could be jointly targeted in multiple health behavior change interventions among early adolescents of diverse backgrounds.

## Introduction

The Centers for Disease Control and Prevention has identified six priority categories of health risk behaviors in youth that contribute to the main causes of morbidity and mortality in this population. These include alcohol and drug use, tobacco use, unhealthy dietary behaviors, and physical inactivity [1]. Many of these behaviors are common among youth and tend to increase with age [1] and continue into adulthood [2], [3]. These risk behaviors do not occur in isolation: there is growing evidence that adolescents tend to adopt sets of ‘healthy’ or ‘unhealthy’ behavior patterns [4]–[6], which is likely to result in a cumulative and detrimental effect on disease risk for segments of the population. Understanding patterns of associations among multiple health behaviors and the extent to which risk behaviors co-occur is essential for informing multiple health behavior screening and interventions [7].

Studies examining multiple health risk behaviors in youth tend to find evidence that risk behaviors co-occur [5], [8]–[11]. In particular, there is evidence that different types of alcohol and other drug (AOD) use tend to cluster [4], [12]. This clustering is often explained in reference to problem-behavior theory, which posits that an underlying behavioral syndrome drives certain youth to adopt multiple problem behaviors and arises from an imbalance of risk factors relative to protective factors across personality and socio-environmental domains [13], [14]. Risk factors that cumulate across individual, social, and environmental domains – that could be conceptualized as an “overall health risk factor” - might also lead subsets of youth to adopt broader sets of unhealthy behaviors. For example, among youth in grades 6 through 12, smoking has been associated with low physical activity and poor diet [5].

Few studies have explored associations among a broad range of youths' health risk behaviors that include substance use, diet, and inactivity, or examined changes in these patterns in early and middle adolescence, a time when these behaviors are being initiated and typically increase. Moreover, although youth with particular individual, social and environmental risk factors are more likely to engage in specific unhealthy behaviors, there is little research that explicitly tests if the clustering of a broad range these behaviors in younger adolescents differs based on demographics such as gender or race/ethnicity.

This study addresses these research gaps by testing for an “overall health risk factor” that results in a broad range of health risk behaviors being positively related and grouping among certain youth. We focus our attention on associations between six behaviors: alcohol use, cigarette smoking, marijuana use, physical inactivity, sedentary computing/gaming time, and the consumption of low-nutrient energy-dense food (fast food, soda). We also add to the extant literature by examining associations among these behaviors in a younger age group that is racially and ethnically diverse. A further aim of the study is to test for differences in the associations among these behaviors as adolescents get older, using longitudinal data, and to examine potential differences based on gender, race/ethnicity, and socioeconomic status (SES). This will allow us to determine if multiple health risk behaviors are more strongly associated over time, and among adolescents with particular socio-demographic attributes. Findings have the potential to provide important information on early trajectories of multiple risk behaviors at a crucial time in the initiation and establishment of lifestyle habits.

## Methods

### Participants and procedure

Data for the current analyses came from two waves of CHOICE, a longitudinal study that started in 2008 as a large cluster randomized trial of a voluntary alcohol and drug intervention for middle school students [15]. Primary intervention outcomes were measured in 2009, with additional survey waves added to better understand how substance use changes over time in this large and diverse sample. The project evaluated the effects of the intervention on adolescent AOD use [15], and questionnaire items on diet, physical activity, and screen time were collected in two later waves (Spring 2010 and Spring 2011).

Participants were recruited from 16 public middle schools across three metropolitan school districts in Southern California. Schools were chosen for their geographic and racial/ethnic diversity. The current analyses focus on participants who completed surveys in Spring 2010 and/or Spring 2011 (which we refer to as Wave 1 and Wave 2 in this paper), resulting in a sample of 8360, of whom 50% were female, 36% were in grade 6, 33% in grade 7, and 31% in grade 8 (at Wave 1). The sample is also unique in its racial/ethnic diversity: 52% Hispanic, 17% Asian, 16% White, and 15% Black/multiethnic/other, which is broadly representative of the ethnic composition of the relevant school populations based on published demographic information for these middle schools in the three school districts at that time. Rates of lifetime and past month substance use in this sample of middle school students are also comparable to national samples [16].

Paper and pencil surveys using a Scantron form were administered during physical education class by trained staff who described the surveys and reviewed confidentiality. Survey booklets were available in English, Spanish, and Korean (just 3–5% of students used the Spanish survey and less than 1% of students used the Korean survey version), and took approximately 45 minutes to complete.

### Ethics statement

Active parental permission was required for the survey and participation in CHOICE. A total of 14,979 students across the 16 schools received parent consent forms: these were returned by 92% of parents and 71% gave permission for their child to participate in the study (*n* = 9,828), which is higher or comparable to other school-based survey completion rates with this population [17]–[19]. A Certificate of Confidentiality was obtained from the National Institutes of Health, and study materials and procedures were approved by the institution's Internal Review Board, the school districts, and the individual schools. Because this is an ongoing study, and as per the IRB requirements and certificate of confidentiality, this dataset cannot be made public.

### Measures

#### Alcohol, cigarette, and marijuana use

Using Monitoring the Future items [18], data were collected on the number of days in the past month that respondents a) consumed one full drink of alcohol, b) used cigarettes, and c) used marijuana (7 point response scale, where 1 = 0 days and 7 = 20 to 30 days). Due to the low prevalence of these behaviors in middle school, dichotomous variables were created, where 1 =  any use and 0 =  no use.

#### Intake of low-nutrient, energy-dense (LNED) foods

Intake of LNED foods was assessed by two items that measured the frequency (number of days in the past 7 days) that respondents “ate fast food” and the number of days they drank regular (non-diet) soda or energy drinks (examples of fast food restaurants and types of non-diet soda and energy drink were provided). These measures have been used in other surveys of middle school youth [1], [20].

#### Physical activity

Participants reported the number of days in the previous week (0 to 7 days) that they were physically active for a total of 60 minutes or more per day. ‘Physical activities’ were defined as activities that increase heart rate and make them breathe hard some of the time. Items were taken from the California Healthy Kids Survey and Youth Risk Behavior Surveillance Survey [1], [20].

#### Sedentary computing and gaming time

Participants reported on the weekly frequency of various sedentary screen-based activities by indicating the number of days in a typical week (0 to 7 days) that they did the following: sent or received email, text or instant messages; visited social networking sites; viewed or posted material online; or played video games. Participants also reported on the amount of time they spent doing each of these activities (1 =  none, 9 =  6 or more hours) on a typical weekday, and on a typical weekend day [1], [20]. These items were used to calculate the typical number of weekly computing/gaming screen time hours. We focused on screen-based activities that included entertainment, social media, and communication, rather than specific school-related activities (e.g., homework, word processing) to capture leisure-time screen usage.

#### Socio-demographics

Socio-demographic measures included age, gender and race/ethnicity. SES was assessed based on parent's education, which has been found to be a valid indicator of socioeconomic position, and is often related to youth risk behaviors [21]. Respondents reported on the highest level of education completed by their father and mother, from four categories (1 =  did not finish high school, 2 =  graduated from high school, 3 =  some college, 4 =  graduated from college). A variable indicating whether at least one parent had graduated from college was created from this information.

### Statistical analyses

Associations among the health behaviors were assessed using confirmatory factor analysis [22], to determine if an underlying health risk latent variable could account for the covariance of multiple health risk behaviors (substance use, intake of LNED foods, physical inactivity, and sedentary screen time). In order to determine the degree to which the behaviors were related, we used composite reliability methods [23], [24]. Composite reliability (CR) is calculated based on the factor loadings (λ) and error variance (ε) for each measured variable in a factor model. For continuous variables CR is given by: 
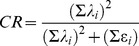
(CR is equivalent to Cronbach's alpha when the assumptions of alpha are satisfied.) For categorical variables, the formula is more complex and we refer the reader to (23). We were interested in evaluating the extent to which CR differed – either between groups of individuals, or within the same group of individuals over time. For each homogenous set of variables identified, at each wave, we calculated CR for each group and time. We therefore calculated separate CRs for males and females (at each wave), separate CRs for those for whom at least one parent attended college and those for whom neither parent attended college, and separate CR for Hispanic, and non-Hispanic respondents. We calculated the difference between the CRs, then used bootstrapping methods (e.g., [25]) to obtain the difference in CR for each group, along with the confidence intervals and significance value. Each estimate used 1000 bootstrap samples. All models were estimated using Mplus 6.11 [26] using WLSMV estimation.

## Results

### Descriptive statistics

Summary statistics of participants' demographics and the seven health behaviors are summarized in Table 1. Similar to our previous findings [27], there was a general trend for substance use to increase as participants increased in age. Of note, the relatively low smoking rates among youth in California have been attributed to well-established anti-smoking prevention programming and public policies [28]. Intake of fast food and soda remained consistent across waves, whereas there was an increase in the number of days per week respondents did 60 minutes or more of physical activity, and an increase in their time spent on the computer and playing video games.

**Table 1:** Descriptive Statistics for Demographic and Health Behavior Variables (N = 8360).

Characteristic		
% male	50.5	
% race/ethnicity		
White (non-Hispanic)	15.8	
Black	3.2	
Hispanic	52.4	
Asian	16.7	
Multi Racial/Other	11.9	
% parent that attended college	62.9	
	**Wave 1**	**Wave 2**
% response rate	92.8	73.3
% any past month alcohol use	5.6	7.3
% any past month cigarette use	1.9	2.6
% any past month marijuana use	3.4	4.8
M (SD) number of days ate fast food in the past week^a^	2.4 (1.5)	2.5 (1.55)
M (SD) number of days drank soda in the past week^a^	2.9 (2.0)	2.9 (2.0)
M (SD) number of days 60 min of activity in the past week^a^	5.2 (2.5)	5.6 (2.4)
M (SD) weekly computer/video game hours^b^	31.8 (25.4)	51.3 (26.7)

a 1 = none, 8 = 7 days

b The hours per week spent doing a specific screen activity (e.g., playing video games, emailing, visiting social networking sites) was computed, and this was summed across all screen activities.

### Confirmatory factor analysis

Longitudinal confirmatory factor analysis [22] was used to assess if the associations among the seven health behaviors could be explained by an underlying health risk latent variable. This hypothesis was not supported as a one factor model had a very poor fit (*χ* ^2^(72) = 2857 CFI = 0.80). A two factor model was found to have a much better fit (Table 2) (*χ* ^2^ (42) = 59 CFI = 1.00), indicating that the associations among these behaviors were better explained by a model with two correlated, yet distinct factors. Although the hypothesized one factor model was not supported, the substance use and health behavior factors were moderately correlated (Wave 1 *r* = 0.33, Wave 2 *r* = 0.32).

**Table 2:** Confirmatory Factor Analysis Estimates and Standard Errors for a Two Factor Longitudinal CFA Model.

	Wave 1			Wave 2		
Factors	Estimate	S.E.	Standar-dized Estimate	Estimate	S.E.	Standar-dized Estimate
*Substance use risk factor*						
Any alcohol	1.004	0.022	0.89	1.010	0.035	0.87
Any cigarettes	1.000*		0.89	1.000*		0.86
Any marijuana	1.035	0.031	0.91	1.081	0.041	0.93
*Health risk factor*						
No of days eat fast food	0.605	0.047	0.64	0.646	0.036	0.70
No of days drink soda	1.000*		0.78	1.000*		0.80
Weekly computer/video hours	0.646	0.043	0.40	0.664	0.044	0.39

*Constrained for identification.

The first factor reflected risk for substance use (alcohol, cigarette, and marijuana use), and the second factor reflected risk for unhealthy eating and sedentary behaviors (fast food intake, soda intake, computing/gaming time). The physical activity variable was dropped from the model as it did not relate to the underlying risk factor as expected (the association was positive meaning that the factor predicted *increased activity* rather than *physical inactivity*).

### Differences in the CR over time, and by demographics: comparing composite reliability

To determine if the substance use factor and the unhealthy eating and sedentary factor has stronger associations over time, composite reliability methods [23], [24] were used to assess differences in the degree to which the groups of variables were related (i.e., the average of the standardized loadings) across waves. As shown in Table 3, reliability for the substance use risk factor was significantly and substantially higher at Wave 2 compared to Wave 1, indicating that alcohol, cigarette, and marijuana use were more closely related at the second time point. There was no statistically significant difference in reliability for the unhealthy eating and sedentary factor over time.

**Table 3:** Composite Reliability Estimates (95%CI) and Difference Tests Across Waves.

	Substance use risk factor	Health behavior risk factor
	Estimate (95% CI)	Estimate (95% CI)
Wave 1	0.21 (0.19, 0.22)	0.60 (0.58, 0.62)
Wave 2	0.46 (0.46, 0.46)	0.62 (0.59, 0.64)
Difference	0.26 (0.24, 0.27), ***p*** **<.001**	0.02 (0.01, 0.04), *p* = .292

Differences in the CR based on participant demographics were also assessed, to understand if there were differences in the degree to which the groups of risk behaviors were related based on gender, parent education, and race/ethnicity. The results, summarized in Table 4, indicate that there were few statistically significant differences in these loadings based on demographic groups within each wave. Based on gender, the CR estimates for the substance use risk factor did not differ significantly between males and females at Wave 1, however the estimate was significantly higher for males (vs. females) at Wave 2 (estimated difference = 0.01, *p* = .001). For the unhealthy eating and sedentary factor, there were no statistically significant differences in composite reliability between males and females at either wave.

**Table 4:** Composite Reliability Estimates (95%CI) and Difference Tests Between Demographic Groups, Within Waves.

Demographic variable	Substance use risk factor		Health behavior risk factor	
	Wave 1	Wave 2	Wave 1	Wave 2
**Gender**				
Male	0.20	0.47	0.60	0.61
	(0.18, 0.22)	(0.46, 0.47)	(0.57, 0.63)	(0.59, 64)
Female	0.21	0.45	0.60	0.61
	(0.19, 0.23)	(0.45, 0.46)	(0.56, 0.62)	(0.58, 0.64)
Difference	0.01	0.01	0.01	0.00
	(−0.02, 0.03)	(0.01, 0.02)	(−0.04, 0.04)	(−0.04, 0.04)
	*p* = .702	*p* = **.001**	*p* = .687	*p* = .965
**Parent Education**				
Attended college	0.19	0.46	0.60	0.62
	(0.17, 0.21)	(0.45, 0.47)	(0.57, 0.63)	(0.59, 0.64)
No college	0.23	0.46	0.60	0.61
	(0.21, 0.24)	(0.45, 0.46)	(0.56, 0.63)	(0.57, 0.64)
Difference	−0.04	0.01	0.00	0.01
	(−0.07, −0.01)	(0.00, 0.01)	(−0.04, 0.04)	(−0.03, 0.05)
	*p* = **.004**	*p* = .233	*p* = .864	*p* = .769
**Ethnicity**				
Hispanic	0.22	0.45	0.60	0.62
	(0.21, 0.24)	(0.44, 0.46)	(0.57, 0.62)	(0.60, 0.65)
Non-Hispanic white	0.22	0.46	0.58	0.58
	(0.18, 0.25)	(0.45, 0.47)	(0.51, 0.62)	(0.52, 0.64)
Difference	0.00	0.01	0.02	0.04
	(−0.0., 0.05)	(0.00, 0.02)	(−0.04, 0.09)	(−0.02, 0.12)
	*p* = .875	*p* = **.021**	*p* = .500	*p* = .229

Differences in CR estimates based on parent education (attended college vs. no college) were only found for the substance use risk factor at Wave 1. In this wave, reliability for this factor was significantly higher for participants whose parents had no college education (vs. those whose parents had a college education) (estimate difference  = −0.04, *p* = .004), indicating that there were stronger associations between the substance use behaviors among youth whose parents had less education. Composite reliability on this factor did not differ significantly between these groups at Wave 2. For the unhealthy eating and sedentary factor, there were no statistically significant differences in CR between parent education groups at either wave.

Differences in CR estimates based on race/ethnicity were only compared between Hispanic and non-Hispanic white participants, due to the smaller size of other race/ethnic groups, which caused convergence problems. For the substance use risk factor, CR did not differ significantly between Hispanic and non-Hispanic white participants at Wave 1, although CR was significantly higher among non-Hispanic white participants at Wave 2 (estimate difference  = 0.01, *p* = .021). For the unhealthy eating and sedentary factor, there were no statistically significant differences in CR between these groups at either wave.

## Discussion

This study tested the hypothesis that a broad range of risky health behaviors – specifically alcohol, cigarette, and marijuana use; physical inactivity; fast food intake; soda intake; and computing/video gaming time – would be positively associated among particular middle school youth, reflecting one underlying health risk factor. The hypothesis was not supported by the confirmatory factor analysis model as these behaviors were better explained by two distinct, although correlated factors: a ‘substance use risk factor’ and an ‘unhealthy eating and sedentary factor’. Nonetheless, the positive association between these two latent risk factors indicates that youth who report substance use are also somewhat more likely to report eating more fast food, drinking soda, and spending more time on computing/gaming activities, supporting previous research showing that middle school youth are prone to adopting sets of multiple health risk behaviors [5], [9], [11]. Thus, prevention programs that target multiple substances, or that target unhealthy eating and excessive screen time, are important for this younger adolescent population. There is likely to be some benefit to addressing substance use, diet, and sedentary activity simultaneously, possibly reducing the risk for multiple health compromising behaviors.

Physical inactivity was the one measured behavior that did not reflect the hypothesized latent variable(s) as expected. Interestingly, the unhealthy eating and sedentary factor was associated with higher levels of physical activity. Other research on associations between diet, physical activity, and sedentary activities among adolescents have been mixed: one review concluded that sedentary screen time is negatively associated with physical activity [8], however other studies have failed to find evidence of a significant association between computer/video game time and physical activity [29]. There is also little evidence of an association between diet (healthy dietary patterns or energy-dense food consumption) and physical inactivity in youth (e.g., [8]). Measuring physical activity and inactivity in youth is challenging because these are complex and often competing behaviors, and there are clear limitations when relying on self-report. It is possible that the items used in this survey, which asked about activities that “make them breathe hard some of the time”, also captured physically unfit youths' experience of relatively mild physical activities. Additional research, preferably using objective measures of movement (e.g., accelerometers) is needed to better understand the association between these behaviors.

We found some evidence that the alcohol, smoking, and drug use behaviors were more highly associated with the substance use risk factor over time, and had a stronger association for males, non-Hispanic whites, and youth whose parents had less education. However, the magnitude of these differences was small and is therefore unlikely to have substantive clinical relevance. Additionally, there was no evidence that the association of these behaviors differed over time or by demographics. As such, interventions seeking to reduce multiple co-occurring risk behaviors should be equally effective for a broad range of youth, across early adolescence.

### Limitations of the Study

It is important to note some limitations of the study. First, we relied on self-report from adolescents for this study, the limitations of which are well-known, although possibly exaggerated for some types of behavior [30]. Much research has shown that self-report among youth is valid when procedures, such as those used in the current study, are implemented (e.g., discussing confidentiality, using Scantron forms for survey answers, having study staff not affiliated with the school collect information) [31]–[33]. In addition, we dichotomized the AOD variables because of the low prevalence of substance use among this younger age group and so we were unable to explore more nuanced associations in AOD use; however, this is an important age to begin to investigate these associations. Finally, because the main focus of the survey was to assess AOD use, there were a limited number of items assessing food intake, physical activity, and sedentary activities. More detailed or objective measures of these behaviors, gathered using daily diaries and accelerometers are preferable, but were not possible to collect in this study.

### Contributions of this study and broader implications

Health compromising behaviors are known to co-occur among adolescents; in particular different types of substance use [5], [9], [11]. There is also some evidence that substance use is positively related to unhealthy eating and physical inactivity, although these findings have been mixed and are typically not tested among a diverse sample of youth [8], [10], [29]. Studies show some evidence that these patterns differ by gender [10], although differences based on other demographics are not known.

This study adds to this literature by exploring longitudinal associations among a broader range of health risk behaviors than has been investigated previously, among a large and racially and ethnically diverse sample of early-middle adolescents. We identified two distinct, yet related groups of associated behaviors that could be jointly targeted in multiple health behavior change interventions among early adolescents. This was also one of the first studies to test if the associations among these behaviors varied over time, or based on gender, race/ethnicity, and socioeconomic status. We found little evidence of any differences.

Given that substance use, unhealthy eating, and computer/video game screen time were found to co-occur, programs could seek to address all three of these behaviors and potentially reduce risk for broader health consequences among this population. Specifically, programs that already target multiple substance use behaviors among at-risk youth are in a position to potentially have an impact on energy-dense food consumption and screen time, with important cost-saving potential. For example, motivational interviewing interventions have an agenda setting component where patients identify a behavior they are ready to change [34]; programs could leverage this opportunity to target co-occurring health risk behaviors.

Early adolescence is a critical time for initiation and escalation of many risk behaviors and can provide an important opportunity for health promotion. Study findings suggest that people that interact with this younger age group, such as teachers, primary care providers, counselors etc. should take the opportunity to address multiple behaviors in both their screening protocols and prevention programming.
